# Negative Impact of Telework, Job Insecurity, and Work–Life Conflict on Employee Behaviour

**DOI:** 10.3390/ijerph20054182

**Published:** 2023-02-26

**Authors:** Marcela-Sefora Nemțeanu, Dan-Cristian Dabija

**Affiliations:** Faculty of Economics and Business Administration, Babeș-Bolyai University, 400570 Cluj-Napoca, Romania

**Keywords:** teleworking, work–life conflict, counterproductive work behaviour, professional isolation, job insecurity, turnover intentions

## Abstract

The COVID-19 pandemic imposed a large-scale adoption of teleworking in various fields, accepted by many employers as the ideal solution to protect their employees against the risk of contracting SARS-CoV-2. Working from home generated substantial savings for organisations and also contributed to alleviating employee stress. In addition to the potential positive effects, telework during COVID-19 favoured counterproductive behaviour, job insecurity, and intention to retire because of the negative outcomes generated by the growing conflict between personal life and working from home and professional and social isolation. The purpose of this research is to define and analyse a conceptual model capable of highlighting the way in which telework, job insecurity, and work–life conflict led to professional isolation and turnover intention, and finally, to the counterproductive behaviour of employees during the COVID-19 pandemic. This research was implemented using employees in Romania, an emerging European economy severely affected by the recent pandemic. The results have been analysed with the help of structural equations in SmartPLS, thus reflecting a significant influence of teleworking on work–life conflict, professional isolation, intentions, and insecurity during the pandemic. The insecurity of employees trained in teleworking contributes significantly to enhancing work–life conflict and professional isolation.

## 1. Introduction

The end of 2019 brought an accelerated spread of the new SARS-CoV-2 virus in China [1], which rapidly expanded in 2020 on a global scale generating a pandemic [2]. High contagion and global spread of the virus engendered swift and radical decision making on a government level, concerning mainly sanitary protection, isolation, the wearing of facial masks, vaccination, and reduced meetings and social activities [3,4,5]. A total of 41.7 million employees worked through teleworking during 2021, doubling the number of teleworkers compared to 2019 [6]. As a result of the COVID-19 pandemic, organisations were forced to turn to teleworking without being prepared [7,8,9], and organisational support, better supervision, and new practices in human resource management adapted to the new context of the pandemic were required [10].

Before the COVID-19 pandemic, teleworking was adopted sporadically in certain industries, such as IT (which by nature allowed remote virtual interactions [11]), education [12], and partially in governance or retail, etc. [13,14,15]. Compared to office work or in-person work, teleworking boasts benefits valued by employees, including no need to commute, more leisure time, better time management, reduced commute costs [16,17], but also challenges, such as professional isolation [18], work–life conflict [19,20], turnover intentions [18,21,22], and even counterproductive work behaviours [8,9,23]. The recent COVID-19 pandemic generated among employees increased job insecurity [24]. This was especially the case in industries where economic activities were significantly reduced or even completely stopped for a time (tourism, air travel, entertainment, cultural services, etc.) [25]. Due to the scarcity of research on the negative implications of teleworking and the increased COVID-19 job insecurity, this research aims to evaluate the way in which teleworking, job insecurity, and work–life conflict led to professional isolation, turnover intentions, and counterproductive work behaviour during the COVID-19 pandemic.

Teleworking is often theorised as related to the resource drain, accommodation, and conservation of resources theory [26,27,28,29,30,31,32]. The paper aims to explain and contribute to these theories, especially regarding the conceptualisations of teleworking dimensions, which exerted a negative influence on employee behaviour. The conceptual model delineated in the literature is subsequently verified using collected data from Romanian employees engaged in teleworking. Romania is an emerging market and was strongly affected by the COVID-19 pandemic, at least in the first waves of its manifestation [33,34] wherein countless citizens lost their lives due to distrust of authorities and/or the virus [35], but also due to phenomena of massive disinformation and widespread fake news [36]. 

The paper is structured as follows: after delineating fundamental theories to understand the phenomenon of teleworking, the negative effects brought about by teleworking are presented, including job insecurity, work–life conflict, turnover intentions, professional isolation, and counterproductive work behaviour. Finally, the conceptual model proposed by the authors is highlighted. The second section outlines the research methodology, the research context, and sample, along with the measurement models and analyses conducted. The fourth part consists of the results from tested the hypotheses through SmartPLS (SmartPLS GmbH, Oststeinbek, Germany) along with a subsequent discussion. The article concludes by highlighting the theoretical and managerial implications and the limitations and future directions of investigation.

## 2. Literature Review

### 2.1. Teleworking and Supporting Managerial Theories

Approaches regarding work undertaken outside the traditional office space are referred to as teleworking [16,37,38,39], remote work [18,39,40], work from home [41,42], or online work [43]. Teleworking has been studied in terms of family life through the lens of managerial theories and psychology. The resource drain theory, for example, is based on the premise that an employee has limited resources of time, attention, and energy. For this reason, these resources will be allocated to the extent in which they do not affect the employee’s family relations or social activities [26]. 

Teleworking has also been approached from the standpoint of accommodation theory [37,44,45], which is based on the premise that the employee will opt out of certain work activities to better meet the needs of the family [46]. Based on this theory, we evaluate the extent to which the pressure of both teleworking and family (as a result of increased work–life conflict) influence employee volition [37] and the extent to which the need for professional development, relational capacities, and autonomy is satisfied in teleworking compared to office work [45].

From the point of the conservation of resource theory, employee well-being is diminished when faced with resource drain [27,28,29,30,31,32]. Work–life conflict can also be explained by the theory of boundaries [47], which opines that if flexibility is a factor, then boundaries can become fuzzy [48]. Teleworking itself presents a situation in which boundaries between work and personal life are made flexible, becoming fuzzy. The various circumstances in which in the employee stays at home interfere with the boundaries imposed by the work schedule, leading to possible conflictual situations or to diminished work productivity [19]. Teleworking was also associated in previous research with an increase in workload and an increase in responsibilities, as they included house duties alongside work tasks [49]. The boundaries between work and family responsibilities become unclear [50]. Teleworkers also have weakened boundaries regarding working hours, co-worker disturbances, and interruptions, which each generate a negative impact on their well-being [51]. Approaching telework from the perspective of the theory of boundaries highlights the impact of work–life conflict and counterproductive work behaviours [52].

### 2.2. Antecedents and the Effects of Telework in a Pandemic Context

The impact of teleworking on personal life has positive effects due to work–life balance but is also less favourable as it exerts relative pressure on household chores. Oftentimes, an employee working from home is forced to dedicate more time to tasks, thus finding difficulty in focussing due to having to switch between household chores [53]. Lack of balance between telework and private life had a significant negative influence on women’s health during the COVID-19 pandemic [54]. The work–life conflict was greater in situations when teachers were forced to telework as a result of COVID-19 quarantine when they had not previously worked remotely [12]. Employees were not prepared for remote working, encountering difficulties in managing the blurring line between personal life and work, requiring more organisation and supervisors to support them [55]. The employee feels domestic tension that decreases their ability to focus [37]. Supervisors have been found to rarely understand the pressure of domestic life generated by family members, by poor management of work, and domestic tasks, while having high expectations about work productivity [56]. Therefore, we conclude that:

**Hypothesis** **1** **(H1).** *Teleworking during the COVID-19 pandemic influences the conflict between work and life.*

Before the COVID-19 pandemic, resorting to short periods of teleworking reduced turnover intention [57]. Teleworking for longer periods may generate dissatisfaction due to the lack of interaction with co-workers and/or supervisors, lack of socialising, a change in scenery, identifying personal development opportunities, etc. [16]. Moreover, extended teleworking diminishes employee performance, favouring professional isolation and affecting their turnover intentions [18,58]. Flexible work arrangements, such as teleworking, do not necessarily reduce the intention to change. Only when employees feel they can benefit from a flexible work arrangement can they make independent decisions about tasks and work activities, thus diminishing turnover intentions. Otherwise, if forced to perform certain tasks and feel that their liberties are fenced, along with their task management and/or decision-making regarding work tasks, then their turnover intention to increases [31]. Therefore, we can conclude that:

**Hypothesis** **2** **(H2).** *Teleworking during the COVID-19 pandemic influences the turnover intention.*

Socioeconomic and sanitary crises favour societal imbalances with direct implications on subsequent stability and job insecurity [59,60,61]. During the COVID-19 pandemic, to allow task performance in safe and sanitary conditions, organisations of all kinds resorted to teleworking to be able to continue work [62,63,64]. Teleworking involves distancing, interrupting direct contact with co-workers and supervisors [65], thus causing stress, insecurity, reduced performance and/or work productivity, reduced personal development opportunities [66,67,68,69], and reduced job security [62]. In fact, unfavourable settings foster employee fear of losing their job. As such crises impact the economy, reducing work activity in certain business sectors led to some job loss [70]. In such contexts, job insecurity increases and employees develop legitimate fears regarding their organisational belonging, etc. [25,71,72]. Therefore, we assess that:

**Hypothesis** **3** **(H3).** *Teleworking influences the insecurity of COVID-19 jobs*.

Professional isolation of employees is a poorly researched topic in the literature, but one that has direct implications on employee well-being. Isolation represents a great organisational challenge for telework. Full- and part-time teleworking arrangements have significant and positive implications on the professional isolation felt by employees daily [38,57]. Isolation from co-workers and/or supervisors diminishes employee job performance [16,18], generating counterproductive behaviours [8,9] and affecting well-being [11]. In teleworking, communication with co-workers decreases and the interaction with supervisors is often limited only to essential aspects [73]. The lack of direct interactions disconnects the employee from relevant information and/or rapid identification of opportunities in professional development. The COVID-19 pandemic also contributed to less face-to-face interaction between colleagues, leading to the need to develop other interactions in order to supply the micro-breaks that help an employee relax [74]. The COVID-19 pandemic context also increased the need of teleworking employees to have constant communication with colleagues and supervisors, as this interaction significantly influenced their well-being [51]. Although employees dedicated more time to tasks, this context favours professional isolation [75]. Therefore, we assess that:

**Hypothesis** **4** **(H4).** *Teleworking during the COVID-19 pandemic influences professional isolation*.

Work–life conflict is a teleworking constant; oftentimes, work-related activities mix with domestic ones [76]. Regardless of gender, age, and marital status, employees with children are less susceptible to teleworking than those without children. Single employees and/or those without children are more prone to teleworking than married employees, and men favour this activity more than women [19]. As the most important asset of family life, children play a vital role in deciding on teleworking. This increases workplace conflicts, family conflict, and work–life conflict, triggering frequent redivision and redistribution of domestic chores among couples [19]. In managing conflicts from their personal lives regarding work, employees may feel pressured; their volition may feel violated, and they may feel isolated [77]. The lack of options to solve these challenges generated by work–life conflict may often escalate these problems [78]. By hindering employees from sharing their experiences with co-workers or supervisors regarding daily struggles to perform and/or be productive and also undertake subsequent household chores, professional isolation becomes an issue. Professional isolation is often marked by lack of professional development, but also by diminished interest in development opportunities and pursuing roles with greater responsibilities [30]. In extreme cases, a successful career is no longer a desideratum for employees. Teleworking causes employees to feel isolated and less trusting in the organisation to which they belong [79], resulting in further distancing from it. Therefore, we evaluate the following relationship:

**Hypothesis** **5** **(H5).** *Work–life conflict generated professional isolation during the COVID-19 pandemic.*

Work–life conflict exerts negative implications not only on employee well-being as a predictor of burnout [80], but also on productivity [81], leading to counterproductive work behaviours. Due to limited time, focus, and effort, the employee may not perform work tasks properly [27,28]. Work–life conflict makes employees feel overwhelmed about their life and their productivity plumets [82]. Work–life conflicts are often associated with increased counterproductive work behaviours [83], thus contributing to decreased organisational engagement [84]. In teleworking, the employee tends to dedicate time to nonwork-related activities during work hours, which favours the dissolution of clear boundaries regarding household and work tasks. Fuzzy boundaries may easily lead to counterproductive work behaviours [52]. Teleworking generates deviant work behaviours, and employees tend not to be aware of office rules, work ethics, and policies pertaining to the workplace. Thus, they tend to compromise by flaunting rules or outright violating them [82]. Of course, exhibiting counterproductive work behaviours depends on emotional intelligence and employee self-discipline regarding the management of work–life conflicts [28]. Therefore, we assess that:

**Hypothesis** **6** **(H6).** *Work–life conflict generated counterproductive work behaviours during the COVID-19 pandemic*.

The recent COVID-19 pandemic amplified the intensity felt by employees about job insecurity, especially in areas where restrictions were more severe [85]. Job insecurity and work–life conflicts influence each other, and their impact is felt more prominently among men [86] as they disturb employee work–life balance and task performance [29]. Stress generated by job insecurity can aggravate work–life conflicts. It leads to emotional exhaustion, as employees try to solve work tasks or tackle them superficially [19], ultimately reducing employee well-being [87,88] and their health [89]. Employees subjected to stress during the pandemic were more prone to depression and anxiety [90]. Therefore, we opine that:

**Hypothesis** **7** **(H7).** *COVID-19 job insecurity worsened work–life conflict.*

Job insecurity is a negative factor in performance; individuals who experience job insecurity exhibit low levels of job satisfaction [8,9], and are thus prone to counterproductive work behaviours [91,92,93], social and/or professional isolation [25,71,72], or turnover intentions [94]. The impact of job insecurity on professional isolation is poorly researched in the pandemic context. Job insecurity engenders anxiety and social isolation among employees [95]. Therefore, the following hypothesis states that:

**Hypothesis** **8** **(H8).** *COVID-19 job insecurity influenced professional isolation.*

Professional isolation is a negative outcome of employee activity in which they no longer foster a sense of belonging to the organisation. Teleworking by nature causes the employee to allocate more and more time to working remotely. The employee disconnects from the community which reduces subsequent interactions with co-workers. The personal and professional isolation experienced influences job performance, but also counterproductive work behaviours [18,25]. Control in teleworking is an issue marked by previous research, which shows that trust between a remote employee and an organisation and its supervisors is an important factor for ensuring productivity [96]. Decreased focus at work based on the lack of interaction with supervisors and monitoring, distraction due to unfettered access to social networks, along with limited interactions with co-workers may contribute to counterproductive behaviours, such as tardiness, neglect of duties, or even poor task performance [23,97,98]. Based on these arguments, we consider the following:

**Hypothesis** **9** **(H9).** *Professional isolation influenced counterproductive work behaviour during the COVID-19 pandemic.*

The employee may intend to leave the organisation due to lack of identification with the organisation’s brand or values or from job dissatisfaction, but also due to other opportunities for career growth [99]. Oftentimes, lacking or toxic leadership can exacerbate turnover intentions, which may lead employees to exhibit unwanted or counterproductive behaviour [100]. Turnover intentions are a detrimental factor favouring counterproductive behaviours [100,101]. Counterproductive work behaviours (deviant activities which may affect the organisation, such as delaying tasks, superficiality, absenteeism, frequent interference with co-workers, hostile behaviour, bullying, etc.) engender the most detrimental of outcomes, which may influence organisational productivity [83]. In teleworking, counterproductive work behaviours may occur as a result of distractions due to unfettered access to social media platforms, widespread activity in the digital environment, and due to decreased interaction with co-workers [102,103,104]. However, such counterproductive behaviours negatively impact organisational well-being and affect individual performance, and may have boomerang effects on other employees [105]. Therefore, we assess that:

**Hypothesis** **10** **(H10).** *Turnover intention influenced counterproductive work behaviour during the COVID-19 pandemic*.

### 2.3. Research Model

**Figure 1:**
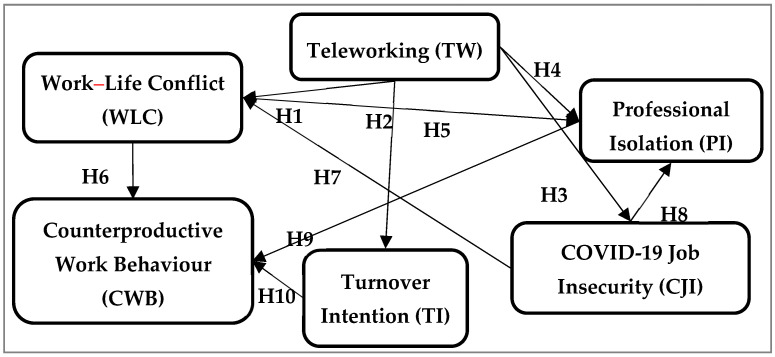
Theoretical model: negative implications of teleworking, job insecurity, and work–life conflict on employee behaviour. Source: own conceptualisation.

Based on these arguments, the conceptual model presented in Figure 1 highlights the links between work–life conflict [19,76], turnover intentions [18], job insecurity, and professional isolation [8,18] in relation to teleworking. Counterproductive work behaviours are amplified by the lack of constant interaction with supervisors [23,97,98], but also by professional isolation and turnover intentions [101].

## 3. Research Methodology

Teleworking in the COVID-19 pandemic was generally employed by European organisations. For instance, the number of employees working remotely in France and England doubled in the spring of 2020 compared with the pre-pandemic period. Australia recorded a 15% increase in teleworking in 2020, while Japan recorded an almost 28% increase. In January and February 2020, teleworkers represented only 5% of the workforce in France, Great Britain, the USA, Italy, Japan, and Sweden [106], since it was employed primarily in the following industries: information and communication (more than 50%), professional, scientific, or technical activities (more than 35%), electricity, gas, steam, and air conditioning (more than 30%), education (over 30%), finance and insurance (more than (more than 20%), and real estate (over 20%) [7]. The proportion of employees working remotely expanded rapidly in educational services [15] and consulting [14], as these business sectors are well suited for digital platforms [13]. In the European Union, teleworking rose by 20% in 2021 compared with 2019, and women were more frequently involved in such activities [6]. In Romania, teleworking was quickly adopted in the COVID-19 pandemic context. Compared with approximately 14,000 teleworking contracts as of 1 January 2020, after 3 months this had increased to approximately 59,000 contracts, and by the end of 2020 the number had risen to 435,000, largely in banking and IT [107]. Nine out of ten employees would prefer teleworking and 60% of respondents would prefer at least a hybrid work arrangement, according to a recent study [108]. Since 2022, Romanian public administration employees are allowed to work remotely 5 days a month [109]. Another effect of COVID-19 and teleworking burnout was the high intention to leave the current job. After the first year of the pandemic, 29.9% of 1000 respondents in a Romanian study declared that in teleworking they started to work earlier, and nearly 40% finished their workday later than in office. A total of 58% of females and 45% of males declared that their stress level increased. Based on burnout and higher levels of stress, the highest level of resignations was registered in Romania in the summer of 2021, with over 40,000 resignations per month peaking in September, which saw over 45,404 resignations [110].

To address the research question, ‘what are the negative implications of teleworking on employee performance?’ and to investigate the links of the conceptual model (Figure 1), we used a quantitative survey in 2021 to examine Romanian employees who turned to teleworking. The questionnaire was developed according to existing scales, using a 5-point Likert scale (total disagreement/total agreement). It comprised of questions regarding work–life conflict [111], teleworking [37], turnover intentions [112], professional isolation [18], job insecurity [8,9], and counterproductive work behaviours [113]. From more than 1500 questionnaires distributed, a total of 641 responses could be obtained. Table 1 illustrates the sociodemographic characteristics of the respondents.

SmartPLS 3.0 [114] calculated the model using the model of the least square-based structural equation and analysed the data in two steps. Firstly, the measurement model was evaluated to determine the reliability and validity of the operational measurements, and then the relationship between the latent structures was validated. To evaluate the effectiveness and reliability of external models, confirmation factors were considered. The results reveal that there is internal consistency of the model (Table 2) because all item loads exceeded 0.7 [115]; Cronbach’s Alpha is higher than 0.7 [116], the average deviation extracted value below 0.5 [117], and the composite reliability value above 0.7 [115]. 

According to the Fornell–Larcker procedure [118], for each latent variable AVE’s value is higher than the correlation coefficient between the competent and all distinct variables (Table 3). Interitem collinearity with the variance inflation factor (VIF) was also tested and the values ranged between 1.477–3.252 (<3.3) [119]. The highest VIF of the inner model was 1.574 (WLC→PI), which indicates no multicollinearity. For hypothesis testing, the bootstrap procedure was applied to assess the relationship between the latent variables. Based on t statistics, the 10 hypothesizes were accepted (Table 4).

Recent research reveals the importance of testing the discriminant validity to determine the degree to which all constructs are indeed distinct. This is known as the HTMT criterion [120]. Therefore, the discriminant validity is indicated by values of HTMT lower than 0.8 [121], as presented in Table 4.

## 4. Results

**Figure 2:**
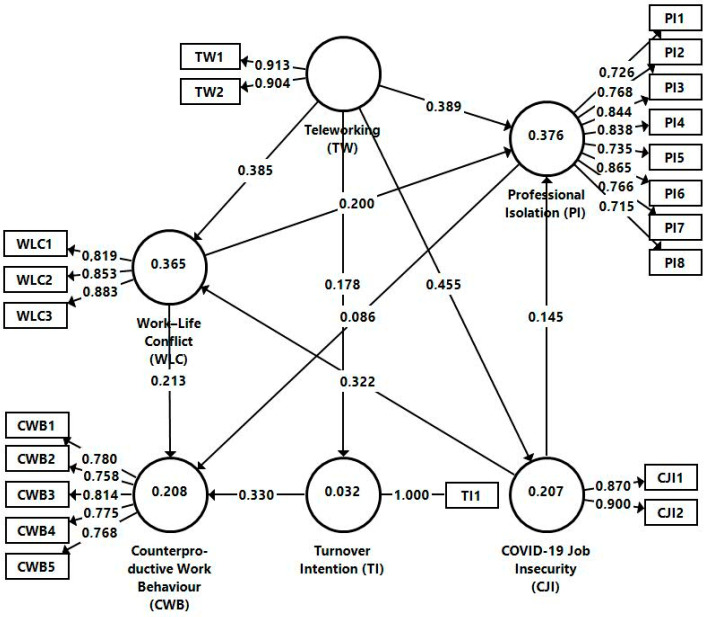
Research model results. Source: research results.

The results of this model are shown in Figure 2. The model’s adjustment statistics show that it is acceptable, and the square root average residual value (SRMR) reaches the recommended threshold at 0.069 < 0.08.

The results of the model are presented in Table 5. The result (β = 0.385; T-value = 10.601; *p* < 0.001) indicates a positive significant effect between teleworking and the conflict between work and home during the COVID-19 pandemic; therefore, H1 can be accepted. This influence is confirmed by the literature [19], which pinpoints the conflict between the pressures of teleworking and domestic responsibilities [37]. The employee must manage household duties that compete with the time and energy allotted for work [53]. H2 assumed that teleworking influences the turnover intention. The results (β = 0.178; T-value = 4.647; *p* < 0.001) illustrate that there is a positive influence on resignation intention; therefore, H2 can be supported. The literature does not prove the significance of the link between teleworking and turnover intention [31], which highlights the originality of this research.

According to the analysis (β = 0.455; T-value = 12.724; *p* < 0.001), teleworking had a strong positive impact on job insecurity during the COVID-19 pandemic, meaning that H3 can be accepted. The authors of [62] reached similar conclusions in the pandemic context. The results of the analysis (β = 0.389; T-value = 8.943; *p* < 0.001) show that teleworking has a strong positive impact on professional isolation, which means that H4 can be accepted. Employee isolation is a direct result of lack of interaction with supervisors and/or co-workers [38], which is a frequent scenario in the pandemic context [73].

Work–life conflict during the COVID-19 pandemic has a weak but significant positive influence on professional isolation (β = 0.200; T-value = 4.360; *p* < 0.001); thus, H5 can be accepted. Although this relation has not been previously tested, it confirms assumptions in the literature which assumed a direct and negative impact of work–life conflict on employee well-being [30,78]. The results (β = 0.213; T-value = 4.909; *p* < 0.001) indicate that the conflict influences counterproductive work behaviour; therefore, H6 is accepted, according to the literature [28,82].

Job insecurity caused by COVID-19 exerts a strong and positive influence (β = 0.322; T-value = 8.393; *p* < 0.001) on work–life conflict, meaning that if employees are stressed because the pandemic could trigger job loss, they tend to feel pressured between work and home responsibilities; therefore, H7 can be accepted. This relationship has not previously been tested in pandemic situations, but the literature presumed the existence of a link between job insecurity and increased work–life conflicts [29,86]. Job insecurity during COVID-19 also generates professional isolation. Job insecurity during COVID-19 had a significant and positive influence on professional isolation (β = 0.145; T-value = 3.456; *p* < 0.010); therefore, H8 can be accepted. The literature [25,71,72] pointed out that job insecurity is linked to employee isolation.

Professional isolation had a positive influence on counterproductive work behaviour during the COVID-19 pandemic according to the results (β = 0.086; T-value = 1.982; *p* < 0.048); thus, H9 can be supported. This relationship has yet to be thoroughly researched in the literature; notwithstanding, teleworking is considered responsible for reduced employee interaction with supervisors and/or co-workers, along with generating counterproductive work behaviours [23,97,98]. Counterproductive work behaviour is also positively influenced by turnover intentions (β = 0.330; T-value = 9.106; *p* < 0.001), so H10 is also validated, as highlighted in the literature [100,101].

## 5. Discussion

Teleworking constitutes an arrangement by which employees perform work-related tasks remotely. The literature highlights the benefits and drawbacks of teleworking [16], but not the negative aspects concerning employee behaviour. Teleworking is associated with increased work–life conflict, as the pressures of work contrast personal responsibilities. The increase in conflict concerning personal life and work is thus explained through the theories of resource drain and accommodation [19].

Before COVID-19, pressure from family members along with the necessity of task performance within teleworking contributed to increased work–life conflicts [37]. The effect of one day of teleworking on the work-to-home conflict is negative (β = −0.60; *p* < 0.01), whereas the effect of one day of teleworking on the home-to-work conflict was positive (β = 0.31; *p* < 0.01); the employee prefers to participate less in work-related tasks to instead undertake domestic responsibilities. Our results (see H1) contradict Metselaar et al. [32], who found that in the pre-pandemic context in the public sector in Portugal, there was a positive link between teleworking and an increase in work–life balance. Our study is also in line with the results of several COVID-19 pandemic-context studies. One study concluded that teleworking generated strain for employees in the form of work–family conflicts due to difficulty of detaching themselves from work at the end of the day. Personal difficulties and remote working contributed to the feeling of professional disconnection between teleworkers and their co-workers and their supervisors [50]. Another study found that work–life conflict was generated because many employees were required to work with children, teenagers, or young adults who were also working from home, generating a difficult management of boundaries which increased conflict [103].

Teleworking contributed to the exacerbation of turnover intentions among employees; the link between constructs is weak but positive and statistically significant. Confronted with teleworking, employees who no longer identify themselves with the organisation and who no longer benefit from the same career development opportunities decide to leave. Our results (see H2) show that teleworking has a positive and strong impact on turnover intentions, which is inconsistent with the literature regarding teleworking from before the pandemic [57], but in accordance with more recent results. This emphasizes that there is an intention to resign among employees working from home in the pandemic context, especially when they no longer identify with the given tasks [31,122].

Teleworking contributes to COVID-19 job insecurity; the link between these dimensions is strong, statistically significant, and positive. Although teleworking was adopted as an alternative solution to in-person work or for the sanitary protection of the employees [63,123], this arrangement contributes to increased job insecurity, an aspect that has been assumed by literature [62]. Adopting teleworking in the COVID-19 context favours an increase in job insecurity (see H3) despite its apparent flexibility, meaning it is no longer considered a positive factor contributing to employee job security.

Teleworking causes interactions with supervisors and/or co-workers to decrease [73], which leads to professional isolation [38]. The existence of some positive influences between working from home and the feeling of professional isolation felt daily by fully remote employees (β = 0.423; T-value = 3.184; *p* < 0.01) was demonstrated, but also for those working partially from home (β = 0.283; T-value = 2.364; *p* < 0.05) [38]. During the COVID-19 pandemic, organisations that adopted teleworking provided a large variety of technological tools for maintaining good communication, but this was not enough to avoid professional isolation [50], social isolation, marginalisation, and other forms of deterioration in the quality of relationships between co-workers and employees [124]. Therefore, professional isolation becomes a negative outcome of telework (see H4).

Work–life conflict generated by teleworking also contributes to increasing professional isolation as our results prove (see H5). Even though the implication of work–life conflict has not been previously tested, studies in the context of COVID-19 indicate several negative implications of work–life conflict and a lack of boundaries, which increases the sense of professional isolation felt by the employee [50]. Interruption and increased conflict with family also have a negative impact on employee well-being [51].

The obtained result (see H6) shows that work–life conflict engenders counterproductive work behaviours, confirming that a lack of focus and distractions from tasks due to a focus on family members lead to counterproductive behaviours [52,82], such as delays in task completion, performing them incorrectly, inefficient communication, etc. The literature [82] demonstrates a strong, direct, and positive link between work–life conflict and counterproductive work behaviour (β = 0.563; T-value = 0.563; *p* < 0.001) among Pakistan’s employees. Jiang et al. [28] show through polynomial regression that there is a direct and significant link (β = 0.353; *p* < 0.001) between work–life conflict and counterproductive behaviours.

The context of the COVID-19 pandemic [125,126] engendered higher levels of job insecurity due to various business sectors becoming halted or restricted [98]. The results (see H7) show that COVID-19 job insecurity leads to increased work–life conflicts. Similar results were highlighted regarding the positive and significant link between job insecurity and work–life conflict [29,86,87].

Job insecurity has negative implications on employee well-being. Unsure of their future in the organisation, employees tend to feel professionally isolated. The link between job insecurity and professional isolation is insufficiently studied in the literature, especially during sanitary crises. The literature highlights the negative implications of job insecurity and social isolation along with professional isolation among employees [25,71,72]. By researching the behaviour of Indian employees working during the lockdown, the authors of [72] prove that there is a positive and significant link (β = 0.242; *p* < 0.001) between job insecurity and isolation caused by teleworking (see H8).

Professional isolation is associated with counterproductive work behaviours (see H9). An employee who feels professionally isolated will have difficulty performing tasks on time or performing them correctly, thus becoming less productive. Counterproductive work behaviours constitute real challenges for organisations that often record delays in project goal achievement or even difficulties in closing them [23]. 

Turnover intentions are associated with counterproductive work behaviours (see H10) as the link between the constructs is positive and significant. Similar results were obtained among Chinese employees who did not participate in teleworking [101]. In their case, the link between the concepts was weak but statistically significant (β = 0.27; *p* < 0.01). In pandemics, there is a more prominent link between these two constructs (β = 0.33; *p* < 0.001), which has been confirmed by the literature [100]. The implications of turnover intentions on counterproductive work behaviours have been scarcely researched in teleworking contexts, especially during pandemics.

## 6. Conclusions

This research contributes to the theory of resource drain, accommodation, resource conservation, social exchange, and self-determination applied to organisational behaviour of employees in the context of teleworking. The paper expands the implications of teleworking as a flexible work arrangement on work outcomes, highlighting the negative effects on both employee well-being and behaviour. This study emphasises the direct impact of teleworking on employee well-being and employee behaviour, such as work–life conflict, turnover intentions, COVID-19 job insecurity, and professional isolation. These negative results have implications at the organisational level, especially in work–life conflict, thus contributing to counterproductive work behaviours and professional isolation. Professional isolation and turnover intentions significantly contribute to an increased incidence of counterproductive work behaviours.

Managerial implications reflect the way COVID-19-related teleworking has negative consequences. This paper acts as a review of some negative implications of this work situation. Work–life conflict, turnover intentions, COVID-19 job insecurity, and professional isolation are directly influenced by teleworking. At the same time, these results influence the increase in counterproductive work behaviour of employees.

Among the limitations of this research is the fact that negative implications of teleworking have been studied at a national level, namely Romania, which calls for an extension of research to other emerging countries, along with comparisons between states that have been strongly affected by teleworking versus those in which teleworking was rather reduced in terms of adoption. Future research could compare the impact of teleworking among employees from various business sectors because it is more suitable for education, consulting, and IT. Teleworking would be an interesting topic for observation in the context of ‘the new normal’ and to analyse this work arrangement in a stable socioeconomic and sanitary setting. Future research could consider expanding the conceptual model by considering the influence of digital platforms and/or tools for work management, handling team communication, and the implications of teleworking on the employee–supervisor relationship.

## Figures and Tables

**Table 1:** Demographic characteristics of the respondents.

Demographics (*n* = 641)		Frequency	Relative Frequency %
Birth year	1950–1979	117	18.3%
	1980–1994	155	24.2%
	1995–2010	369	57.6%
Gender	Male	245	38.2%
	Female	396	61.8%
Education level	High school diploma	150	23.4%
	Bachelor and master’s degree	491	76.6%
Net monthly income	No income	50	7.8%
	Up to 269 €	45	7.0%
	269–636 €	271	42.3%
	636–1272 €	217	33.9%
	Over 1272 €	58	9.0%

Source: own results.

**Table 2:** Scale reliability.

Construct	Item	Measurement	Loading	Cronbach’s Alpha	AVE	CR
Work–Life Conflict [111]	WLC1	My work during the COVID-19 pandemic affected my relationship with my spouse/partner and/or with my children.	0.819	0.811	0.726	0.888
	WLC2	My work during the COVID-19 pandemic influenced my level of stress.	0.853			
	WLC3	My work during the COVID-19 pandemic affected my domestic ability to balance my work responsibilities.	0.883			
Teleworking [37]	TW1	I felt pressure from my employer to use teleworking practises.	0.913	0.788	0.825	0.904
	TW2	I felt pressure from my family to use teleworking practises.	0.904			
Turnover intention [112]	RI1	I intend to stay in my profession, but to leave my current organisation at or before the end of this year.	1.000	1.000	1.000	1.000
Professional Isolation [18]	PI1	I felt lost in the learning opportunities of others.	0.726	0.911	0.615	0.927
	PI2	Teleworking made me feel lonely.	0.768			
	PI3	Telework kept me away from others.	0.844			
	PI4	I did not have direct contact with other colleagues, face-to-face.	0.838			
	PI5	Working at home made me feel isolated.	0.735			
	PI6	I missed my colleagues’ emotional support.	0.865			
	PI7	I missed the informal interactions with others.	0.766			
	PI8	I felt like I lost the opportunity to learn from others.	0.715			
COVID-19 Job Insecurity [8,9]	CJI1	I felt directly affected by my job.	0.870	0.725	0.783	0.879
	CJI2	I felt insecure about my job.	0.900			
Counterproductive Behaviour [113]	CWB1	I complained about things that were not important at work.	0.780	0.839	0.607	0.885
	CWB2	I made the problems bigger than the ones they were working on.	0.758			
	CWB3	I focused on the negative aspect of the working situation rather than the positive aspect.	0.814			
	CWB4	I discussed the negative aspects of my work with colleagues.	0.775			
	CWB5	I spoke to people outside the organisation about the negative aspects of my work.	0.768			

Note: CJI: COVID-19 job insecurity; CWB: counterproductive work behaviour; PI: professional isolation; teleworking (TW); turnover intention (TI); work–life conflict (WLC). Factor loading > 0.7; Cronbach’s Alpha > 0.7; average variance extracted (AVE) > 0.5; composite reliability (CR) > 0.7. Source: own results.

**Table 3:** Discriminant validity analyses.

Construct	CJI	CWB	PI	TW	TI	WLC
CJI	0.885					
CWB	0.173	0.779				
PI	0.422	0.229	0.784			
TW	0.455	0.366	0.562	0.908		
RI	0.110	0.373	0.124	0.178	1.000	
WLC	0.498	0.304	0.479	0.531	0.150	0.852

Note: CJI: COVID-19 job insecurity; CWB: counterproductive work behaviour; PI: professional isolation; teleworking (TW); turnover intention (TI); work–life conflict (WLC). Source: own results.

**Table 4:** Heterotrait–monotrait ratio (HTMT).

Construct	CJI	CWB	PI	TW	TI	WLC
CJI						
CWB	0.222					
PI	0.494	0.244				
TW	0.598	0.452	0.640			
RI	0.129	0.399	0.121	0.199		
WLC	0.651	0.368	0.530	0.664	0.167	

Note: CJI: COVID-19 job insecurity; CWB: counterproductive work behaviour; PI: professional isolation; teleworking (TW); turnover intention (TI); work–life conflict (WLC). Source: own results.

**Table 5:** Path coefficients of the structural equation model.

Paths	Path Coefficients	Standard Deviation	T-Value	*p*-Value	Hypotheses
TW→WLC	0.385	0.036	10.601	0.000 ***	H1-Confirmed
TW→RI	0.178	0.038	4.674	0.000 ***	H2-Confirmed
TW→CJI	0.455	0.036	12.724	0.000 ***	H3-Confirmed
TW→PI	0.389	0.044	8.943	0.000 ***	H4-Confirmed
WLC→PI	0.200	0.046	4.360	0.000 ***	H5-Confirmed
WLC→CWB	0.213	0.043	4.909	0.000 ***	H6-Confirmed
CJI→WLC	0.322	0.038	8.393	0.000 ***	H7-Confirmed
CJI→PI	0.145	0.042	3.456	0.001 ***	H8-Confirmed
PI→CWB	0.086	0.043	1.982	0.048 **	H9-Confirmed
TI→CWB	0.330	0.036	9.106	0.000 ***	H10-Confirmed

Note: ** *p* < 0.05; *** *p* < 0.001; Note: CJI: COVID-19 job insecurity; CWB: counterproductive work behaviour; PI: professional isolation; teleworking (TW); turnover intention (TI); work –life conflict (WLC).

## Data Availability

Data available on request.

## References

[1] Harapan H., Itoh N., Yufika A., Winardi W., Keam S., Te H., Megawati D., Hayati Z., Wagner A.L., Mudatsir M. (2020). Coronavirus disease 2019 (COVID-19): A literature review. J. Infect. Public Health.

[2] Pak A., Adegboye O.A., Adekunle A.I., Rahman K.M., McBryde E.S., Eisen D.P. (2020). Economic Consequences of the COVID-19 Outbreak: The Need for Epidemic Preparedness. Front. Public Health.

[3] Nemțeanu M.S., Dinu V., Dabija D.C. (2021). Job Insecurity, job instability and job Satisfaction in the Context of COVID 19 Pandemic. J. Compet..

[4] Papp S., Kimmerl K., Gatz J., Laue M., Grunow R., Kaspari O. (2020). Evaluation of sporicidal disinfectants for the disinfection of personal protective equipment during biological hazards. Health Secur..

[5] Bruinen de Bruin Y., Lequarre A.-S., McCourt J., Clevestig P., Pigazzani F., Jeddi M.Z., Colosio C., Goulart M. (2020). Initial impacts of global risk mitigation measures taken during the combatting of the COVID-19 pandemic. Saf. Sci..

[6] Adascalitei D., Vacas-Soriano C., Staffa E., Hurle J. (2022). Telework and Telework Ability during COVID: An Analysis Using LFS Data. Eurofound. https://www.eurofound.europa.eu/sites/default/files/wpef21041.pdf.

[7] OECD Teleworking in the COVID-19 Pandemic: Trends and Prospects 2021. https://read.oecd-ilibrary.org/view/?ref=1108_1108540-p249kho0iu&title=Teleworking-in-the-COVID-19-pandemic-Trends-and-prospects.

[8] Nemțeanu M.S., Dabija D.C., Stanca L. (2021). The Influence of Teleworking on Performance and Employees’ Counterproductive Behaviour. Amfiteatru Econ..

[9] Nemțeanu M.S., Dinu V., Pop R.A., Dabija D.C. (2022). Predicting Job Satisfaction and Work Engagement Behavior in the COVID-19 Pandemic: A Conservation of Resources Theory Approach. EM Econ. Manag..

[10] van Engen M., Peters P., van de Water F. (2023). Perceived Lockdown Intensity, Work-Family Conflict and Work Engagement: The Importance of Family Supportive Supervisor Behaviour During the COVID-19 Crisis. Virtual Management and the New Normal: New Perspectives on HRM and Leadership since the COVID-19 Pandemic.

[11] Contreras F., Baykal E., Abid G. (2020). E-Leadership and Teleworking in Times of COVID-19 and Beyond: What We Know and Where Do We Go. Front. Psychol..

[12] Santa-Cruz-Espinoza H., Chávez-Ventura G., Domínguez-Vergara J., Merino-Soto C. (2023). Internal Structure of the Work–Family Conflict Questionnaire (WFCQ) in Teacher Teleworking. Int. J. Environ. Res. Public Health.

[13] Hoti K., Jakupi A., Hetemi D. (2020). Provision of community pharmacy services during COVID-19 pandemic: A cross sectional study of community pharmacists’ experiences with preventative measures and sources of information. Int. J. Clin. Pharm..

[14] Situmorang D.D.B. (2020). Online/Cyber Counseling Services in the COVID-19 Outbreak: Are They Really New?. J. Pastor. Care Couns..

[15] Pandit D., Agrawal S. (2022). Exploring Challenges of Online Education in COVID Times. FIIB Bus. Rev..

[16] Baruch Y. (2000). Teleworking: Benefits and pitfalls as perceived by professionals and managers. New Technol. Work. Empl..

[17] Johnson L.C., Andrey C., Shaw S.M. (2007). Dithers Comes to Dinner: Telework and the merging of women’s work and home domains in Canada. Gend. Place Cult..

[18] Golden T.D., Veiga J.F., Dino R.N. (2008). The impact of professional isolation on teleworker job performance and turnover intentions: Does time spent teleworking, interacting face-to-face, or having access to communication-enhancing technology matter?. J. Appl. Psychol..

[19] Zhang S., Moeckel R., Moreno A.T., Shuai B., Gao J. (2020). A work-life conflict perspective on telework. Transp. Res. Part A Policy Pract..

[20] Vyas L., Butakhieo N. (2021). The impact of working from home during COVID-19 on work and life domains: An exploratory study on Hong Kong. Policy Des. Pract..

[21] Bratu S. (2020). The Fake News Sociology of COVID-19 Pandemic Fear: Dangerously Inaccurate Beliefs, Emotional Contagion, and Conspiracy Ideation. Linguist. Philos. Investig..

[22] Ciobanu A., Androniceanu A., Lăzăroiu G. (2019). An Integrated Psycho-Sociological Perspective on Public Employees’ Motivation and Performance. Front. Psychol..

[23] Holland S.J., Simpson K.M., Dalal R.S., Vega R.P. (2016). I can’t steal from a coworker if I work from home: Conceptual and measurement-related issues associated with studying counterproductive work behavior in a telework setting. Hum. Perform..

[24] Shoss M., Van Hootegem A., Selenko E., De Witte H. (2022). The job insecurity of others: On the role of perceived national job insecurity during the COVID-19 pandemic. Econ. Ind. Democr..

[25] Ganson K.T., Tsai A.C., Weiser S.D., Benabou S.E., Nagata J.M. (2021). Job Insecurity and Symptoms of Anxiety and Depression Among U.S. Young Adults During COVID-19. J. Adolesc. Health.

[26] Frone M.R., Quick J.C., Tetrick L.E. (2003). Work-family balance. Handbook of Occupational Health Psychology.

[27] Jiang L., Lavaysse L.M. (2018). Cognitive and affective job insecurity: A meta-analysis and a primary study. J. Manag..

[28] Jiang D., Chen Q., Ning L., Liu Q. (2022). Work-Family Conflict and Counterproductive Behavior of Employees in Workplaces in China: Polynomial Regression and Response Surface Analysis. J. Asian Financ. Econ. Bus..

[29] Nauman S., Zheng C., Naseer S. (2020). Job insecurity and work–family conflict: A moderated mediation model of perceived organizational justice, emotional exhaustion and work withdrawal. Int. J. Confl. Manag..

[30] Ghislieri C., Molino M., Dolce V., Sanseverino D., Presutti M. (2021). Work-family conflict during the Covid-19 pandemic: Teleworking of administrative and technical staff in healthcare. An Italian study. Med. De Lav..

[31] Tsen M.K., Gu M., Tan C.M., Goh S.K. (2021). Effect of Flexible Work Arrangements on Turnover Intention: Does Job Independence Matter?. Int. J. Sociol..

[32] Metselaar S.A., den Dulk L., Vermeeren B. (2022). Teleworking at Different Locations Outside the Office: Consequences for Perceived Performance and the Mediating Role of Autonomy and Work-Life Balance Satisfaction. Rev. Public Pers. Adm..

[33] Nemțeanu M.S., Dabija D.C., Pamfilie R., Dinu V., Tăchiciu L., Pleșea D., Vasiliu C. (2020). Best Practices of Nongovernmental Organisations in Combatting COVID-19. Proceedings of the 6th BASIQ International Conference on New Trends in Sustainable Business and Consumption.

[34] Tecău A.S., Constantin C.P., Lixandroiu R.C., Chitu I.B., Bratucu G. (2020). Impact of the COVID-19 Crisis on Heavy Work Investment in Romania. Amfiteatru Econ..

[35] Marcău F.-C., Purec S., Niculescu G. (2022). Study on the Refusal of Vaccination against COVID-19 in Romania. Vaccines.

[36] Palade I., Balaban D.C. (2020). An Analysis of COVID-19—Related Fake News from Romania. A Pilot Qualitative Study. J. Media Res..

[37] Delanoeije J., Verbruggen M. (2019). The Use of Work-Home Practices and Work-Home Conflict: Examining the Role of Volition and Perceived Pressure in a Multi-Method Study. Front. Psychol..

[38] de Vries H., Tummers L., Bekkers V. (2019). The Benefits of Teleworking in the Public Sector: Reality or Rhetoric?. Rev. Public Pers. Adm..

[39] Stiles J. (2020). Strategic niche management in transition pathways: Telework advocacy as groundwork for an incremental transformation. Environ. Innov. Soc. Transit..

[40] Golden T.D., Eddleston K.A. (2020). Is there a price telecommuters pay? Examining the relationship between telecommuting and objective career success. J. Vocat. Behav..

[41] Giménez-Nadal J.I., Molina J.A., Velilla J. (2020). Work time and well-being for workers at home: Evidence from the American Time Use Survey. Int. J. Manpow..

[42] Sarbu M. (2015). Determinants of Work-at-Home Arrangements for German Employees. Labour.

[43] Grant C.A., Wallace L.M., Spurgeon P.C., Tramontano C., Charalampous M. (2019). Construction and initial validation of the E-Work Life Scale to measure remote e-working. Empl. Relat..

[44] Deci E., Vansteenkiste M. (2004). Self-determination theory and basic need satisfaction: Understanding human development in positive psychology. Ric. Di Psicol..

[45] Brunelle E., Fortin J.-A. (2021). Distance Makes the Heart Grow Fonder: An Examination of Teleworkers’ and Office Workers’ Job Satisfaction Through the Lens of Self-Determination Theory. Sage Open.

[46] Lambert S.J. (1990). Processes linking work and family: A critical review and research agenda. Hum. Relat..

[47] Piszczek M.M., Berg P. (2014). Expanding the boundaries of boundary theory: Regulative institutions and work–family role management. Hum. Relat..

[48] Ashforth B.E., Keiner G.E., Fugate M. (2000). All In a Day’s Work: Boundaries and Micro Role Transitions. Acad. Manag. Rev..

[49] Vinueza-Cabezas A., Osejo-Taco G., Unda-López A., Paz C., Hidalgo-Andrade P. (2022). A Comparison of Working Conditions and Workers’ Perceptions among On-Site, Telework, and Hybrid Workers in Ecuador during the COVID-19 Pandemic. Int. J. Environ. Res. Public Health.

[50] Ivasciuc I.S., Epuran G., Vuță D.R., Tescașiu B. (2022). Telework Implications on Work-Life Balance, Productivity, and Health of Different Generations of Romanian Employees. Sustainability.

[51] Petcu M.A., Sobolevschi-David M.I., Crețu R.F., Curea S.C., Hristea A.M., Oancea-Negescu M.D., Tutui D. (2023). Telework: A Social and Emotional Perspective of the Impact on Employees’ Wellbeing in the COVID-19 Pandemic. Int. J. Environ. Res. Public Health.

[52] Becker W.J., Belkin L.Y., Tuskey S.E., Conroy S.A. (2022). Surviving remotely: How job control and loneliness during a forced shift to remote work impacted employee work behaviors and well-being. Hum. Resour. Manag..

[53] Dockery A.M., Bawa S. (2018). When two worlds collude: Working from home and family functioning in Australia. Int. Labour Rev..

[54] Sygit-Kowalkowska E., Piotrowski A., Boe O., Rawat S., Minic J., Predoiu A., Predoiu R., Vazne Ž., Fernate A., Malinauskas R. (2022). Evaluation of Work Mode and Its Importance for Home–Work and Work–Home Relationships: The Role of Resilience, Coping with Stress, and Passion for Work. Int. J. Environ. Res. Public Health.

[55] Errichiello L., Pianese T. (2021). The role of organizational support in effective remote work implementation in the Post-COVID era. Handbook of Research on Remote Work and Worker Well-Being in the Post-COVID-19 Era.

[56] Lembrechts L., Zanoni P., Verbruggen M. (2018). The impact of team characteristics on the supervisor’s attitude towards telework: A mixed-method study. Int. J. Hum. Resour. Manag..

[57] Caillier J.G. (2013). Are Teleworkers Less Likely to Report Leave Intentions in the United States Federal Government Than Non-Teleworkers Are?. Am. Rev. Public Adm..

[58] Kliestik T., Nagy M., Valaskova K. (2023). Global Value Chains and Industry 4.0 in the Context of Lean Workplaces for Enhancing Company Performance and Its Comprehension via the Digital Readiness and Expertise of Workforce in the V4 Nations. Mathematics.

[59] Wilson J.M., Lee J., Fitzgerald H.N., Oosterhoff B., Sevi B., Shook N.J. (2020). Job Insecurity and Financial Concern During the COVID-19 Pandemic Are Associated with Worse Mental Health. J. Occup. Environ. Med..

[60] Elshaer I.A., Azazz A.M.S. (2022). Amid the COVID-19 Pandemic, Unethical Behavior in the Name of the Company: The Role of Job Insecurity, Job Embeddedness, and Turnover Intention. Int. J. Environ. Res. Public Health.

[61] Mihalca L., Lucia Ratiu L., Brendea G., Metz D., Dragan M., Dobre F. (2021). Exhaustion while teleworking during COVID-19: A moderated-mediation model of role clarity, self-efficacy, and task interdependence. Oeconomia Copernic..

[62] Imran M.A., Ahmed I. (2020). Job Insecurity in Private Education Sector Considering COVID-19 Pandemic: Bangladesh Panorama. Am. Int. J. Bus. Manag. Stud..

[63] Busu M., Gyorgy A. (2021). The Mediating Role of the Ability to Adapt to Teleworking to Increase the Organizational Performance. Amfiteatru Econ..

[64] Mura L., Zsigmond T., Machová R. (2021). The effects of emotional intelligence and ethics of SME employees on knowledge sharing in Central-European countries. Oeconomia Copernic..

[65] Schall M.C., Chen P. (2021). Evidence-Based Strategies for Improving Occupational Safety and Health Among Teleworkers During and After the Coronavirus Pandemic. Hum. Factors.

[66] Clark A. (2020). COVID-19-related Misinformation: Fabricated and Unverified Content on Social Media. Anal. Metaphys..

[67] Lăzăroiu G., Adams C. (2020). Viral Panic and Contagious Fear in Scary Times: The Proliferation of COVID-19 Misinformation and Fake News. Anal. Metaphys..

[68] Sheares G., Miklencicova R., Grupac M. (2020). The Viral Power of Fake News: Subjective Social Insecurity, COVID-19 Damaging Misinformation, and Baseless Conspiracy Theories. Linguist. Philos. Investig..

[69] Tavares F., Santos E., Diogo A., Ratten V. (2021). Teleworking in Portuguese communities during the COVID-19 pandemic. J. Enterprising Communities People Places Glob. Econ..

[70] Burke R.J., Ng E.S.W., Wolpin J. (2014). Economic austerity and healthcare restructuring: Correlates and consequences of nursing job insecurity. Int. J. Hum. Resour. Manag..

[71] Di D. (2021). Surviving is Succeeding: How Tech Workers Handle Job Insecurity During COVID-19. Am. Behav. Sci..

[72] Mehta P. (2022). Work alienation as a mediator between work from home-related isolation, loss of task identity and job insecurity amid the COVID-19 pandemic. Int. J. Workplace Health Manag..

[73] Park S., Cho Y.J. (2022). Does telework status affect the behavior and perception of supervisors? Examining task behavior and perception in the telework context. Int. J. Hum. Resour. Manag..

[74] Junça-Silva A. (2023). Unleashing the Furr-Recovery Method: Interacting with Pets in Teleworking Replenishes the Self’s Regulatory Resources: Evidence from a Daily-Diary Study. Int. J. Environ. Res. Public Health.

[75] Carillo K., Cachat-Rosset G., Marsan J., Saba T., Klarsfeld A. (2021). Adjusting to epidemic-induced telework empirical insights from teleworkers in France. Eur. J. Inf. Syst..

[76] Palumbo R., Flamini G., Gnan L., Pellegrini M.M., Petrolo D., Fakhar Manesh M. (2022). Disentangling the implications of teleworking on work–life balance: A serial mediation analysis through motivation and satisfaction. J. Organ. Eff. People Perform..

[77] Catană S.-A., Toma S.-G., Imbrișcă-Burcea M. (2022). Teleworking Impact on Wellbeing and Productivity: A Cluster Analysis of the Romanian Graduate Employees. Front. Psychol..

[78] Andrade C., Petiz Lousã E. (2021). Telework and Work–Family Conflict during COVID-19 Lockdown in Portugal: The Influence of Job-Related Factors. Adm. Sci..

[79] Donnelly R., Johns J. (2021). Recontextualising remote working and its HRM in the digital economy: An integrated framework for theory and practice. Int. J. Hum. Resour. Manag..

[80] Buonomo I., Fiorilli C., Romano L., Benevene P. (2020). The Roles of Work-Life Conflict and Gender in the Relationship between Workplace Bullying and Personal Burnout. A Study on Italian School Principals. Int. J. Environ. Res. Public Health.

[81] An J., Liu Y., Sun Y., Liu C. (2020). Impact of Work–Family Conflict, Job Stress and Job Satisfaction on Seafarer Performance. Int. J. Environ. Res. Public Health.

[82] Amalia D.P., Zakiy M. (2021). Working Period as a Moderating Variable of Work Family Conflict, Work Stress, and Turnover Intention on Counterproductive Work Behaviour. Perisai Islam. Bank. Financ. J..

[83] Selvarajan T.T., Singh B., Cloninger P.A., Misra K. (2019). Work–Family Conflict and Counterproductive Work Behaviors: Moderating Role of Regulatory Focus and Mediating Role of Affect. Organ. Manag. J..

[84] Kang M., Hong Y., Suh Y. (2018). The effect of work life conflict on organizational commitment and counterproductive work behaviors: The mediating effect of resource loss and negative emotion. Korean J. Ind. Organ. Psychol..

[85] Aguiar-Quintana T., Nguyen T.H.H., Araujo-Cabrera Y., Sanabria-Díaz J.M. (2021). Do job insecurity, anxiety and depression cause by the COVID-19 pandemic influence hotel employees’ self-rated task performance? The moderating role of employee resilience. Int. J. Hosp. Manag..

[86] Richter A., Näswall K., Lindfors P., Sverke M. (2015). Job insecurity and work-family conflict in teachers in Sweden: Examining their relations with longitudinal cross-lagged modeling. PsyCh J..

[87] Buonocore F., Russo M., Ferrara M. (2015). Work–family conflict and job insecurity: Are workers from different generations experiencing true differences?. Community Work. Fam..

[88] Hu S., Jiang L., Probst T.M., Liu M. (2021). The relationship between qualitative job insecurity and subjective well-being in Chinese employees: The role of work–family conflict and work centrality. Econ. Ind. Democr..

[89] Minnotte K.L., Yucel D. (2018). Work–Family Conflict, Job Insecurity, and Health Outcomes Among US Workers. Soc. Indic. Res..

[90] Mirko A., Ruiz-Zorrilla P., Sanz-Vergel A.I., Leon-Perez J.M., Rodriguez-Muñoz A. (2022). The role of job insecurity and work-family conflict on mental health evolution during COVID-19 lockdown. Eur. J. Work. Organ. Psychol..

[91] Probst T.M., Stewart S.M., Gruys M.L., Tierney B.W. (2007). Productivity, counterproductivity and creativity: The ups and downs of job insecurity. J. Occup. Organ. Psychol..

[92] Van den Broeck A., Sulea C., Vander Elst T., Fischmann G., Iliescu D., De Witte H. (2014). The mediating role of psychological needs in the relation between qualitative job insecurity and counterproductive work behavior. Career Dev. Int..

[93] Ma B., Liu S., Lassleben H., Ma G. (2019). The relationships between job insecurity, psychological contract breach and counterproductive workplace behavior: Does employment status matter?. Pers. Rev..

[94] Mauno S., De Cuyper N., Tolvanen A., Kinnunen U., Mäkikangas A. (2014). Occupational well-being as a mediator between job insecurity and turnover intention: Findings at the individual and work department levels. Eur. J. Work. Organ. Psychol..

[95] Obrenovic B., Du J., Godinic D., Baslom M., Majdy M., Tsoy D. (2021). The Threat of COVID-19 and Job Insecurity Impact on Depression and Anxiety: An Empirical Study in the USA. Front. Psychol..

[96] Pianese T., Errichiello L., da Cunha J.V. (2022). Organizational control in the context of remote working: A synthesis of empirical findings and a research agenda. Eur. Manag. Rev..

[97] Kaplan S., Engelsted L., Lei X., Lockwood K. (2018). Unpackaging Manager Mistrust in Allowing Telework: Comparing and Integrating Theoretical Perspectives. J. Bus. Psychol..

[98] Aguinis H., Burgi-Tian J. (2021). Talent management challenges during COVID-19 and beyond: Performance management to the rescue. BRQ Bus. Res. Q..

[99] Kim H., Kim E.G. (2021). A meta-analysis on predictors of turnover intention of hospital nurses in South Korea (2000–2020). Nurs. Open.

[100] Hattab S., Wirawan H., Salam R., Daswati D., Niswaty R. (2022). The effect of toxic leadership on turnover intention and counterproductive work behaviour in Indonesia public organisations. Int. J. Public Sect. Manag..

[101] Xiong R., Wen Y. (2020). Employees’ turnover intention and behavioral outcomes: The role of work engagement. Soc. Behav. Personal. Int. J..

[102] Vega R.P., Anderson A.J., Kaplan S.A. (2015). A Within-Person Examination of the Effects of Telework. J. Bus. Psychol..

[103] Chambel M.J., Carvalho V.S., Santos A. (2022). Telework during COVID-19: Effects on the Work–Family Relationship and Well-Being in a Quasi-Field Experiment. Sustainability.

[104] Chambel M.J., Carvalho V.S., Carvalho A., Machado C., Davim J.P. (2022). Reinventing the Workplace: The Adoption of Telework in Post-COVID Times. Organizational Management in Post Pandemic Crisis. Management and Industrial Engineering.

[105] Bruk-Lee V., Spector P.E. (2006). The social stressors-counterproductive work behaviors link: Are conflicts with supervisors and coworkers the same?. J. Occup. Health Psychol..

[106] Ker D., Montagnier P., Spiezia V. (2021). Measuring Teleworks in the COVID-19 Pandemic.

[107] Radu C. Pandemia a Normalizat Munca de Oriunde. România se Îndreaptă Spre Jumătate de Milion de Angajați Care Lucrează Oficial din Telemuncă. 2022 Economedia.ro. https://economedia.ro/pandemia-a-normalizat-munca-de-oriunde-romania-se-indreapta-spre-jumatate-de-milion-de-angajati-care-lucreaza-oficial-din-telemunca.html#.Y11YbHZBzIV.

[108] Wall-Street (2022). 9 din 10 Angajați Preferă Telemunca, Deși 60% nu au Condiții Bune de Lucru Acasă. https://www.wall-street.ro/articol/Companii/287153/9-din-10-angajatiprefera-telemunca-desi-60-nu-auconditii-bune-de-lucru-acasa.html#gref.

[109] Marina G. (2022). Bugetarii pot Lucra în Telemuncă Cinci Zile pe Lună. https://www.digi24.ro/stiri/actualitate/bugetarii-pot-lucra-in-telemunca-cinci-zile-pe-luna-2120339.

[110] Dimulescu V. (2022). Cum Mai Arată Munca în Pandemie. https://www.scena9.ro/article/muncapandemie-2022.

[111] Hill E.J., Ferris M., Martinson V. (2003). Does it matter where you work? A comparison of how three work venues (traditional office, virtual office, and home office) influence aspects of work and personal/family life. J. Vocat. Behav..

[112] Luna-Arocas R., Camps J. (2007). A model of high-performance work practices and turnover intentions. Pers. Rev..

[113] Koopmans L., Bernaards C.M., Hildebrandt V.H., van Buuren S., van der Beek A.J., de Vet H.C.W. (2013). Development of an individual work performance questionnaire. Int. J. Product. Perform. Manag..

[114] Ringle C.M., Wende S., Becker J.-M. SmartPLS 3. 2015. Boenningstedt: SmartPLS GmbH. https://www.smartpls.com.

[115] Hair J.F., Black W.C., Babin B.J. (2010). Multivariate Data Analysis: A Global Perspective.

[116] Henseler J., Sarstedt M. (2013). Goodness-of-fit indices for partial least squares path modeling. Comput. Stat..

[117] Chin W.W. (1998). The Partial Least Squares Approach for Structural Equation Modeling, Modern Methods for Business Research.

[118] Fornell C., Larcker D.F. (1981). Evaluating structural equation models with unobservable and measurement error. J. Mark. Res..

[119] Diamantopoulos A., Siguaw J.A. (2006). Formative versus reflective indicators in organizational measure development: A comparison and empirical illustration. Br. J. Manag..

[120] Franke G., Sarstedt M. (2019). Heuristics versus statistics in discriminant validity testing: A comparison of four procedures. Internet Res..

[121] Henseler J., Ringle C.M., Sarstedt M. (2015). A new criterion for assessing discriminant validity in variance-based structural equation modeling. J. Acad. Mark. Sci..

[122] Alshaabani A., Oláh J., Popp J., Zaien S. (2020). Impact of Distributive Justice on the Trust Climate Among Middle Eastern Employees. Pol. J. Manag. Stud..

[123] Nemteanu M.S., Dabija D.C. (2021). The Influence of Internal Marketing and Job Satisfaction on Task Performance and Counterproductive Work Behavior in an Emerging Market during the COVID-19 Pandemic. Int. J. Environ. Res. Public Health.

[124] Vayre É., Morin-Messabel C., Cros F., Maillot A.-S., Odin N. (2022). Benefits and Risks of Teleworking from Home: The Teleworkers’ Point of View. Information.

[125] Birtus M., Lăzăroiu G. (2021). The Neurobehavioral Economics of the COVID-19 Pandemic: Consumer Cognition, Perception, Sentiment, Choice, and Decision-Making. Anal. Metaphys..

[126] Lăzăroiu G., Horak J., Valaskova K. (2020). Scaring Ourselves to Death in the Time of COVID-19: Pandemic Awareness, Virus Anxiety, and Contagious Fear. Linguist. Philos. Investig..

